# Data and performance profiles applying an adaptive truncation criterion, within linesearch-based truncated Newton methods, in large scale nonconvex optimization

**DOI:** 10.1016/j.dib.2018.01.012

**Published:** 2018-01-20

**Authors:** Andrea Caliciotti, Giovanni Fasano, Stephen G. Nash, Massimo Roma

**Affiliations:** a Dipartimento di Ingegneria Informatica, Automatica e Gestionale “A. Ruberti”, SAPIENZA, Università di Roma, via Ariosto, 25, 00185 Roma, Italy; b Department of Management, University Ca' Foscari of Venice, S. Giobbe, Cannaregio 873, 30121 Venice, Italy; c Systems Engineering & Operations Research Department, George Mason University, 4400 University Drive Fairfax, VA 22030, USA

## Abstract

In this paper, we report data and experiments related to the research article entitled “An adaptive truncation criterion, for linesearch-based truncated Newton methods in large scale nonconvex optimization” by Caliciotti et al. [1]. In particular, in Caliciotti et al. [1], large scale unconstrained optimization problems are considered by applying linesearch-based truncated Newton methods. In this framework, a key point is the reduction of the number of inner iterations needed, at each outer iteration, to approximately solving the Newton equation. A novel adaptive truncation criterion is introduced in Caliciotti et al. [1] to this aim. Here, we report the details concerning numerical experiences over a commonly used test set, namely CUTEst (Gould et al., 2015) [2]. Moreover, comparisons are reported in terms of *performance profiles* (Dolan and Moré, 2002) [3], adopting different parameters settings. Finally, our linesearch-based scheme is compared with a renowned trust region method, namely TRON (Lin and Moré, 1999) [4].

**Specifications Table**

**Table:** 

Subject area	*Operations Research and Management Science*
More specific subject area	*Nonlinear Optimization*
Type of data	*Table, graph*
How data was acquired	http://www.cuter.rl.ac.uk/*, experimental output data*
Data format	*Raw and filtered*
Experimental factors	*None*
Experimental features	*Different codes have been experienced over the CUTEst test set; then, comparisons among their performance are provided in terms of performance profiles*
Data accessibility	Test problems available at http://www.cuter.rl.ac.uk/. Complete output data available at request to the authors

**Value of the data**

• Output data reported represent a significant benchmark for future comparisons, among different algorithms for large scale unconstrained optimization.
• Output data may be used by other researchers for tuning novel strategies, within truncated Newton methods.
• Output data illuminate the comparison between the linesearch and the trust region approaches, as globalization methods.

## 1 Data

Data from different experimental settings are reported, along with performance profiles, which highlight the advantages of adopting the proposal in [1]. The use of the performance profiles [3] is typically advised in the community of Nonlinear Optimization, since they clearly summarize in one plot the comparison among several codes over an entire test set. We obtain such profiles after filtering the test set from CUTEst collection, in order to guarantee a fair comparison among different codes. In particular, for any test problem, we state that a code fails in solving such a problem whenever *(i)* a given stopping criterion is not satisfied within 100,000 outer iterations, or *(ii)* if the CPU time exceeds 900 s. Moreover, in comparing any two algorithms, we consider only those problems where the algorithms converge to the same stationary point. This is checked by using the test (see [5])

$$\left|{\;f}_{1}^{*}-f_{2}^{*}\right|\leq 10^{-3}\underset{\;}{\min}\left\{\left|{\;f}_{1}^{*}\right|,\left|{\;f}_{2}^{*}\right|\;\right\}+10^{-6},$$

being $f_{1}^{*}$, $f_{2}^{*}$ the optimal function values obtained by the two algorithms. Finally, we discarded all the test problems where the compared algorithms required a CPU time below 0.1 s to solve them.

## 2 Experimental design, materials and methods

In order to assess the Adaptive Truncation Criterion proposed in [1] (named ATC), we consider a standard implementation of a truncated Newton method, namely the linesearch-based truncated scheme described in [6]. Inner iterations are performed using the Conjugate Gradient (CG) method. The novel criterion ATC is adopted in order to avoid over solving of the Newton equation at each outer iteration. In the ATC scheme (see [1]) the maximum number of CG inner iterations allowed at k-th outer iteration (${\max\mathrm{\_it}}_{k}$) is initialized to n, and then adaptively adjusted according to ATC. As regards the parameters in the ATC scheme, we set $\gamma_{1}=10^{-4}$, $\gamma_{2}=10^{-2}$, $\sigma_{1}=2$, $\sigma_{2}=1.1$, $\sigma_{3}=0.2$, $\theta_{1}=10^{-2}$, $\theta_{2}=10^{-4}$.

This choice is suggested by a preliminary coarse tuning on the chosen test set. Moreover, since we tested ATC both within the unpreconditioned and the preconditioned framework proposed in [6], the value of the parameter l is set to 7, in order to allow the construction of an effective preconditioner (see also the discussion about the choice of the parameter $h_{\max}$ in [6]).

The algorithms were coded in FORTRAN 90 and the GFortran compiler under Linux Ubuntu 14.04 was used. The stopping criterion for the outer iterations is the standard one given by

$$||g_{k}||\leq 10^{-5}\underset{\;}{\max}\left\{1,||x_{k}||\right\},$$

where $x_{k}$ denotes the k-th iterate, $g_{k}$ indicates the gradient of the objective function at $x_{k}$ and ‖∙‖ stands for the Euclidean norm.

As regards the set of test problems, we selected all the unconstrained convex and nonconvex large problems available in the CUTEst collection [2], and when a problem is of variable dimension, we considered two different dimensions (usually 1000 and 10,000 variables). The resulting test set consists in 112 problems.

As regards the stopping criterion for the CG inner iterations, we tested both the criteria reported in Section 2 of [1]:

a) *the residual-based criterion*;
b) *the quadratic model reduction-based criterion*.

Since the criterion *a)* with

$$\eta_{k}=\underset{\;}{\min}\left\{\frac{1}{k},||g_{k}||\right\}$$

proved to yield poorer performance in practice, we preferred to use the more reliable residual-based criterion adopted in [6]. This criterion sets

$$\eta_{k}=\underset{\;}{\max}\{||g_{k}||,\sqrt[{3}]{\left||g_{k}|\right|}\}\;\underset{\;}{\min}\left\{\frac{\sqrt{n}}{k},||g_{k}||\right\},$$

which both takes into account the size n of the problem and allows a coarser solution when far from a stationary point. The criterion *b)* adopts $\eta_{k}=0.5,$ as suggested in [7].

In the sequel we adopt the following terminology:

• *ATC-true* stands for algorithms which use the ATC scheme;
• *ATC-false* stands for algorithms which do not use the ATC scheme.

### 2.1 Choice of $C_{k}$ in the ATC scheme

Two different formulae were adopted for the parameter $C_{k}$ in [1]:

(1) $$C_{k}=\underset{\;}{\min}\left\{1,\;\left|f\left(x_{k}\right)\right|\right\};$$

(2) $$C_{k}=\underset{\;}{\max}\{1,\left|f(x_{k})\right|\}.$$

**Fig. 1:**
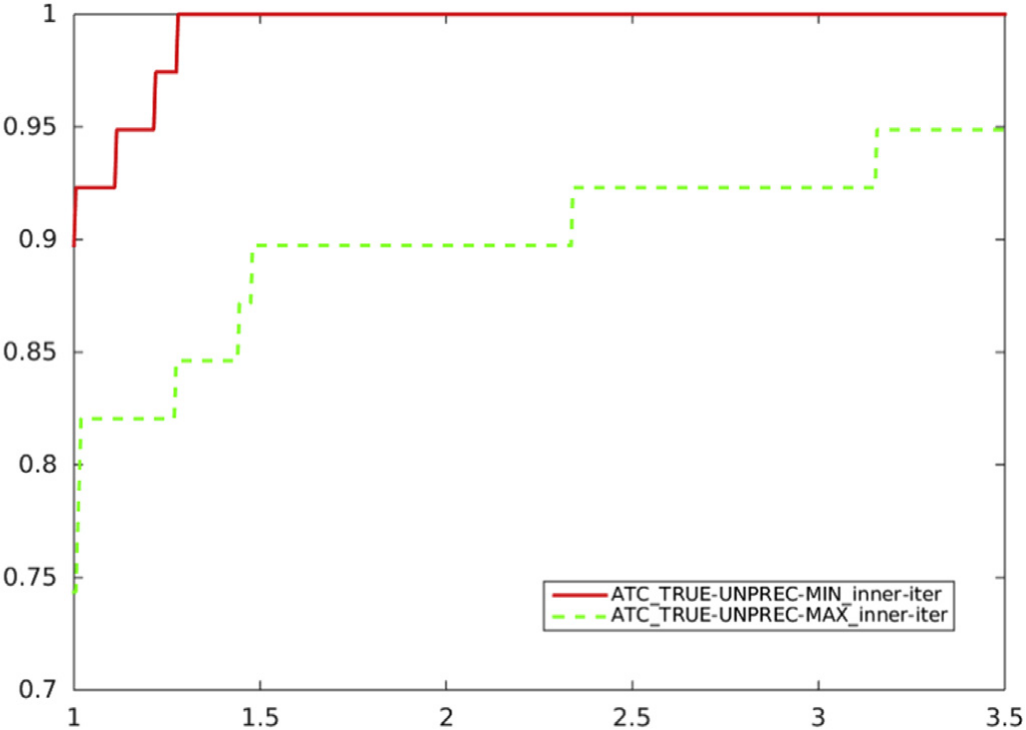
Unpreconditioned truncated Newton method using the residual-based criterion a) with ATC-true: the choice of $C_{k}$ in (1) (solid line) vs. the choice of $C_{k}$ in (2) (dashed line), in terms of CG inner iterations.

**Fig. 2:**
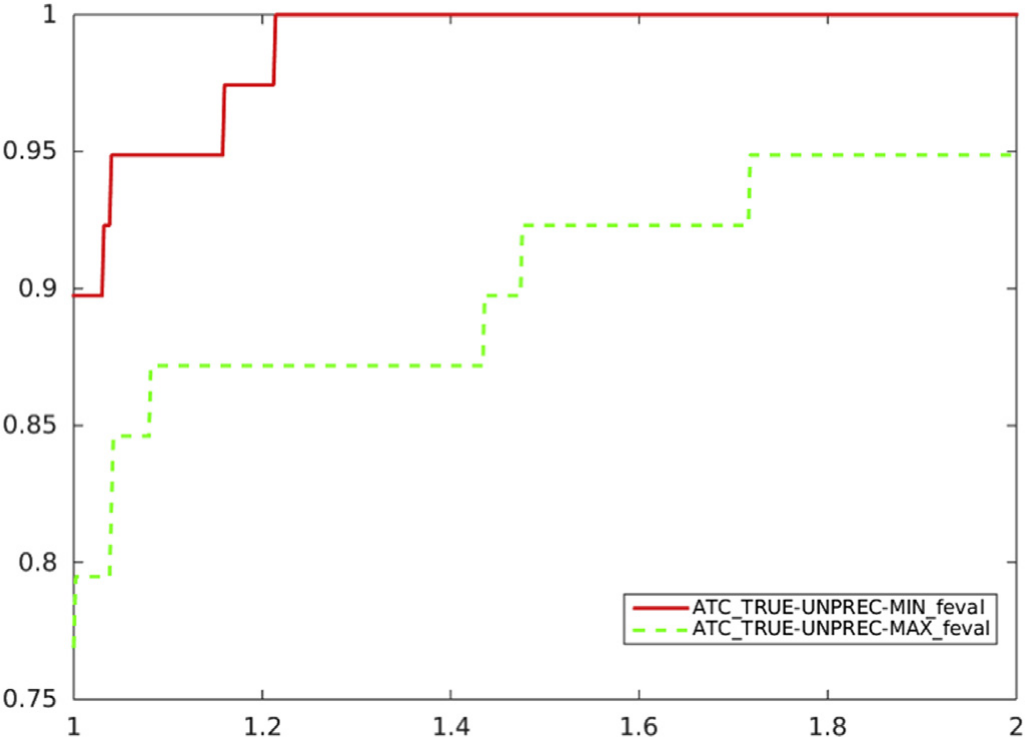
Unpreconditioned truncated Newton method using the residual-based criterion a) with ATC-true: the choice of $C_{k}$ in (1) (solid line) vs. the choice of $C_{k}$ in (2) (dashed line), in terms of function evaluations.

**Fig. 3:**
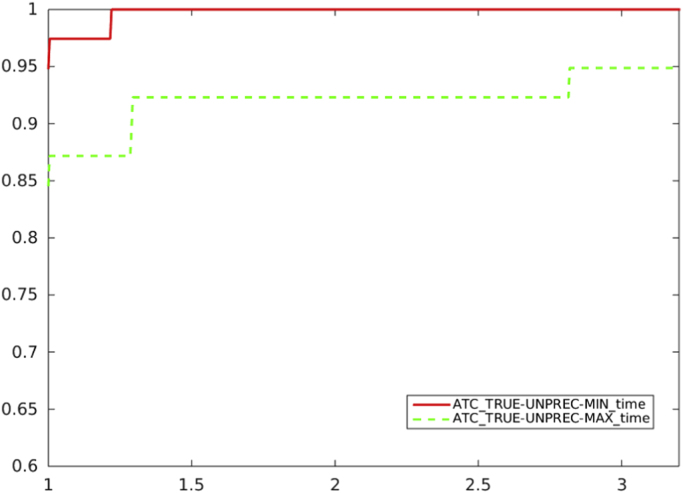
Unpreconditioned truncated Newton method using the residual-based criterion a) with ATC-true: the choice of $C_{k}$ in (1) (solid line) vs. the choice of $C_{k}$ in (2) (dashed line), in terms of CPU time.

Fig. 1, Fig. 2, Fig. 3 report performance profiles of the comparison among schemes where our proposal is adopted, with the two choices (1) and (2) for $C_{k}$.

### 2.2 Numerical comparisons among different truncated Newton schemes

**Fig. 4:**
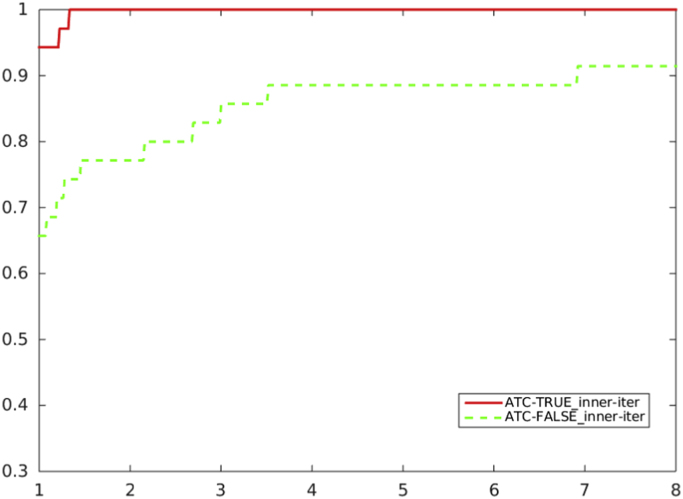
Unpreconditioned truncated Newton method using the residual-based criterion a): comparison ATC-true vs. ATC-false, in terms of CG inner iterations.

**Fig. 5:**
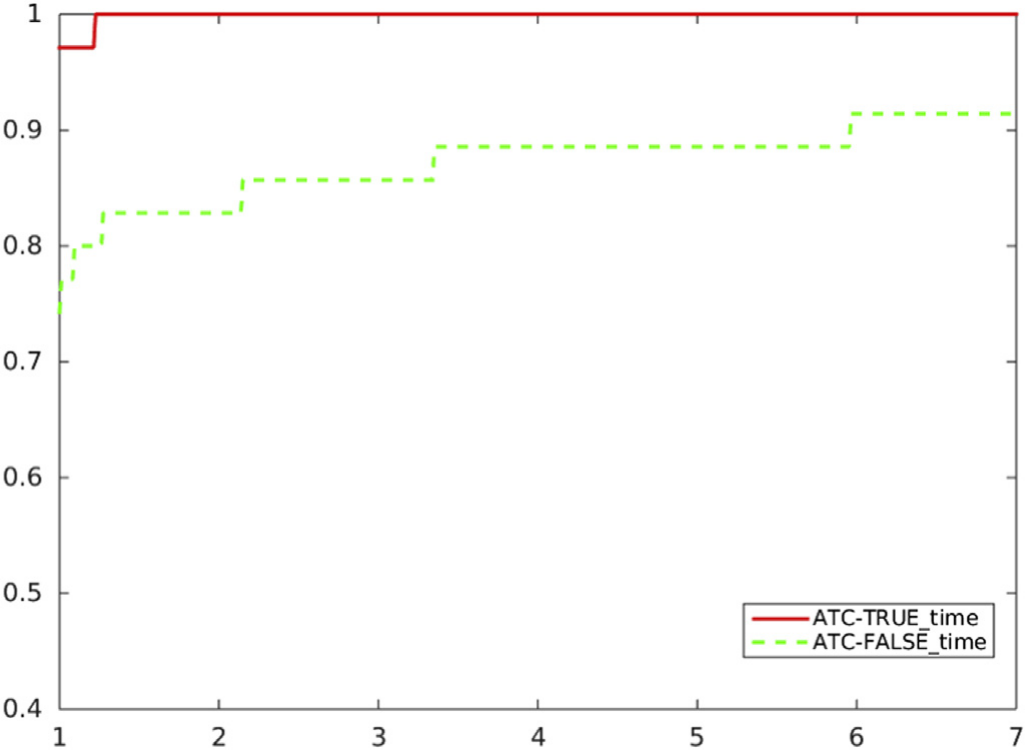
Unpreconditioned truncated Newton method using the residual-based criterion a): comparison ATC-true vs. ATC-false, in terms CPU time.

**Fig. 6:**
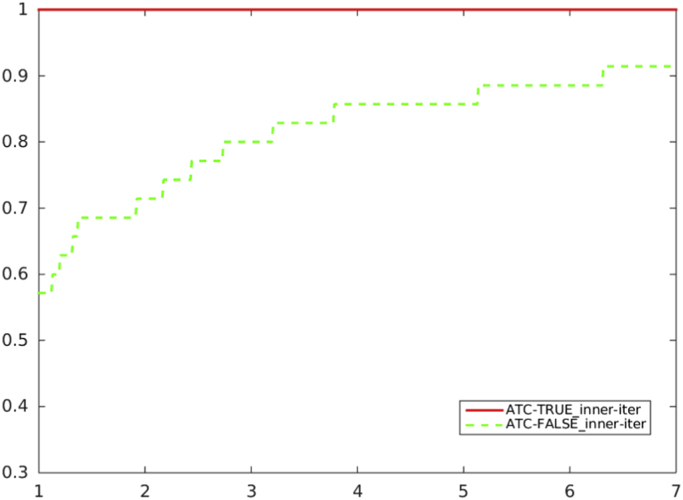
Preconditioned truncated Newton method using the residual-based criterion a): comparison ATC-true vs. ATC-false, in terms of CG inner iterations.

**Fig. 7:**
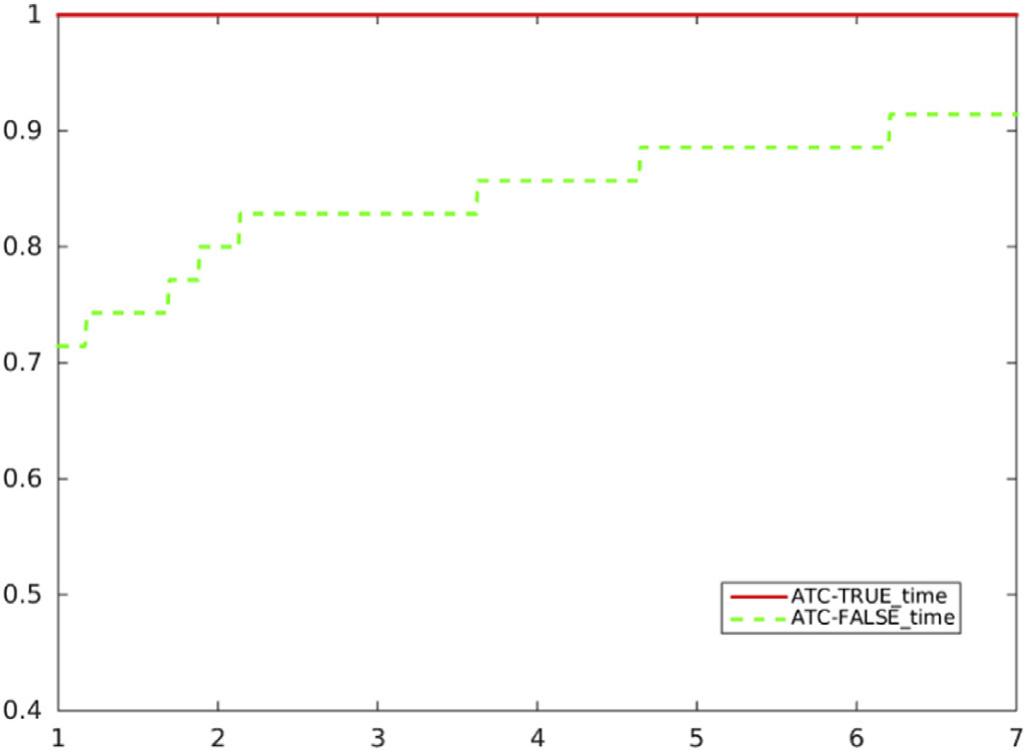
Preconditioned truncated Newton method using the residual-based criterion a): comparison ATC-true vs. ATC-false, in terms CPU time.

Fig. 4, Fig. 5, Fig. 6, Fig. 7 report performance profiles of the comparison between the two algorithmic choices *ATC-true* vs. *ATC-false*, where the residual-based criterion *a)* is adopted in the unpreconditioned and preconditioned cases.

**Fig. 8:**
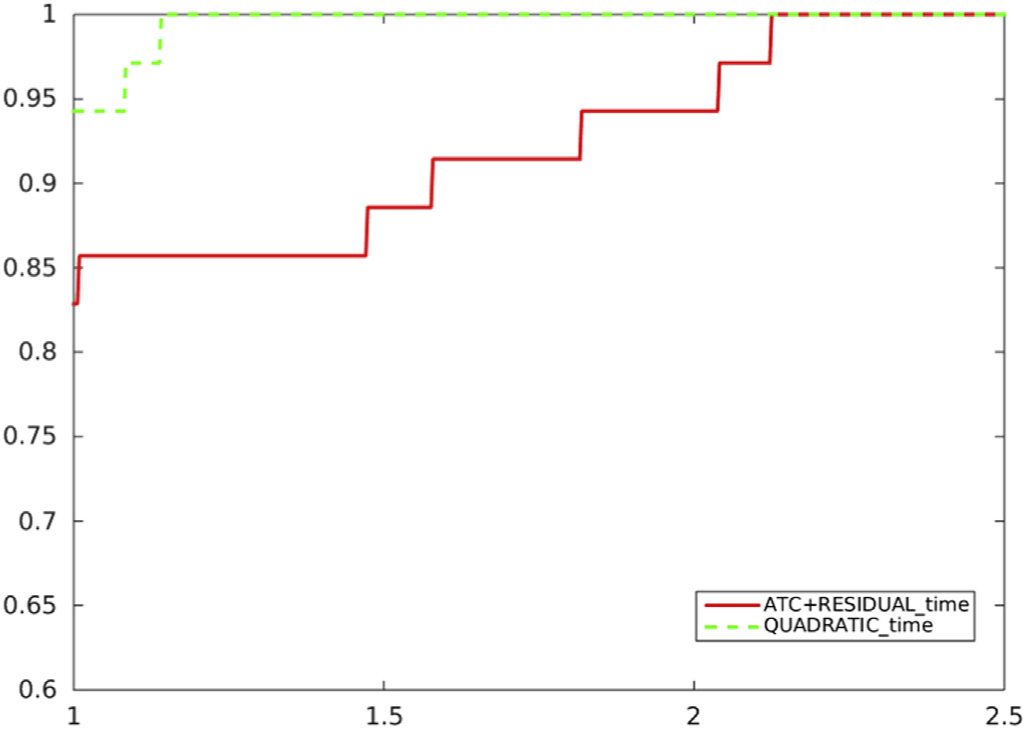
Unpreconditioned truncated Newton method: comparison between the residual-based criterion a) with ATC-true and the quadratic model reduction-based criterion b), in terms of CPU time.

**Fig. 9:**
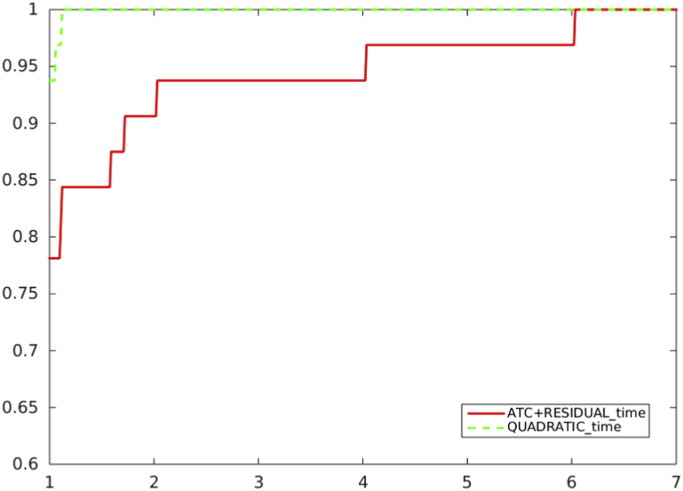
Preconditioned truncated Newton method: comparison between the residual-based criterion a) with ATC-true and the quadratic model reduction-based criterion b), in terms of CPU time.

Fig. 8, Fig. 9 refer to the comparison, in terms of CPU time, between the adoption of the residual-based criterion a*)* and the quadratic model reduction-based criterion *b)* in the algorithm which uses ACT in the unpreconditioned and preconditioned cases.

### 2.3 Comparison with a trust region approach

**Fig. 10:**
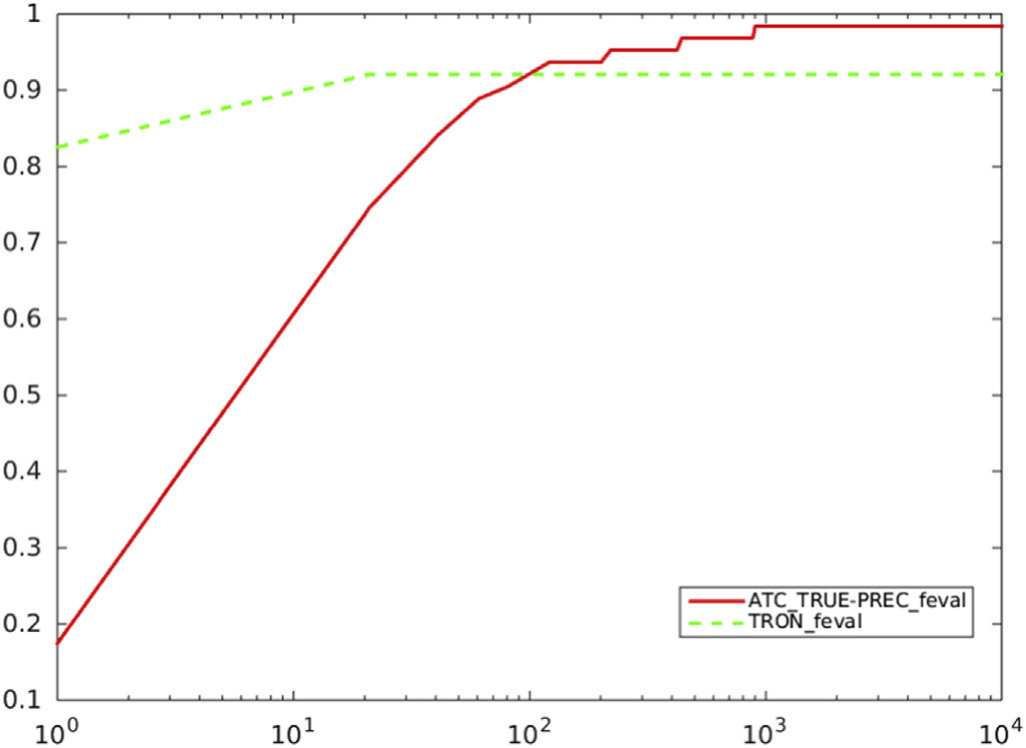
Comparison between preconditioned truncated Newton method with the residual-based criterion a) and ATC-true vs. TRON, in terms of number of function evaluations. Abscissa axis is in logarithmic scale.

**Fig. 11:**
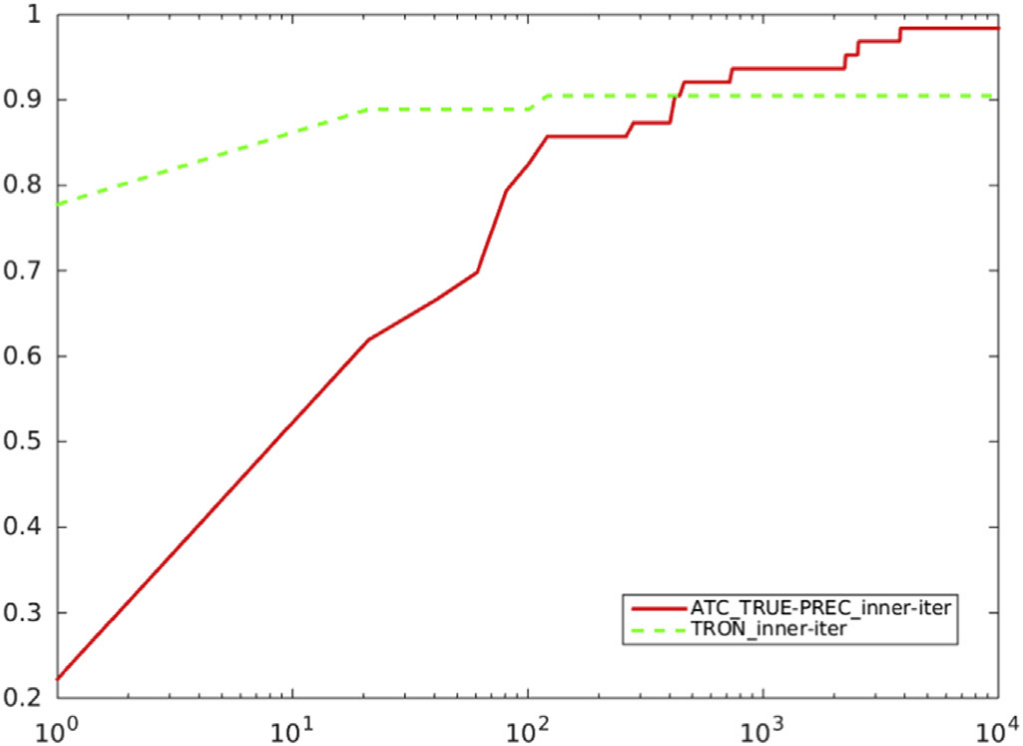
Comparison between preconditioned truncated Newton method with the residual-based criterion a) and ATC-true vs. TRON, in terms of CG inner iterations. Abscissa axis is in logarithmic scale.

**Fig. 12:**
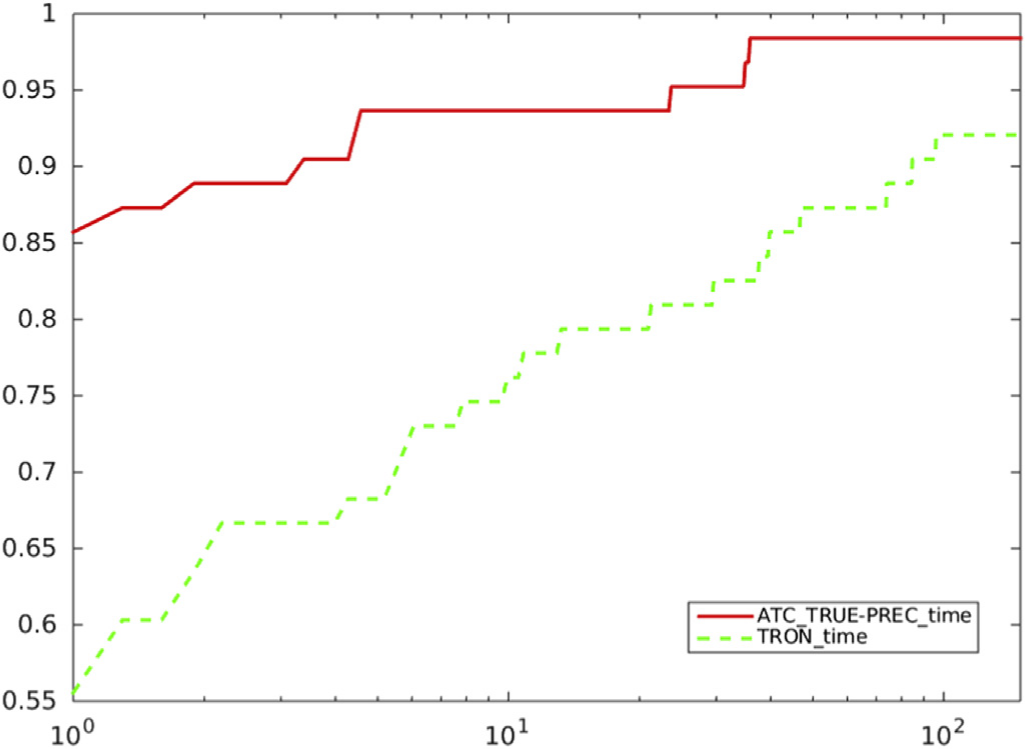
Comparison between Preconditioned truncated Newton method with criterion a), and ATC-true vs. TRON, in terms of CPU time. Abscissa axis is in logarithmic scale.

Fig. 10, Fig. 11, Fig. 12 report performance profiles of the comparison between our proposal of a truncated Newton method, where ATC is adopted (*ATC-true*), and the trust region-based code TRON [4].

Table 1 reports comparisons among the outputs of different versions of TRON and our proposals, on a selection of test problems.

**Table 1:** This table reports the detailed output for all the problems where at least one of the algorithms fails to converge. On problem FLETCBV3 the algorithms converge towards different points, so that the outputs obtained are not comparable.

		TRON			TRON			Precondioned truncated Newton				Precondioned truncated Newton			
		*With the stopping criterion*			*With the stopping criterion*			*With* ATC-true *and*				*With* ATC-false *and*			
		$||g_{k}||\leq 10^{-5}$			$||g_{k}||\leq 10^{-5}\underset{\;}{\max}\left\{1,||x_{k}||\right\}$			$||g_{k}||\leq 10^{-5}\underset{\;}{\max}\left\{1,||x_{k}||\right\}$				$||g_{k}||\leq 10^{-5}\underset{\;}{\max}\left\{1,||x_{k}||\right\}$			
PROBLEM	*n*	it/nf	CG-it	time	it/nf	CG-it	time	it	nf	CG-it	time	it	nf	CG-it	time
FLETCBV3	1000	$>10^{-5}<\mathrm{FigureObject}>$	–	–	9	8	0.00	9	9	14	0.00	9	9	14	0.00
FLETCBV3	10,000	$>10^{-5}$	–	–	1870	1869	10.68	143	143	227	0.45	136	136	176	0.40
MINSURF	5625	–	–	> 900	–	–	> 900	157	361	8414	12.51	23	133	16,160	23.61
NONCVXUN	10,000	–	–	> 900	10,234	16,976	461.02	3072	11,940	25,843	78.61	–	–	–	> 900
PENALTY1	10,000	–	–	> 900	–	–	> 900	64	123	80	0.13	64	123	80	0.11
POWER	10,000	–	–	> 900	–	–	> 900	222	816	13,343	6.03	118	704	84,216	37.38
SINQUAD	10,000	25	36	0.19	25	36	0.19	–	–	–	> 900	–	–	–	> 900
SPARSINE	10,000	–	–	> 900	1999	3,026,104	864.60	901	2562	84,553	144.26	–	–	–	> 900
VARDIM	10,000	–	–	> 900	–	–	> 900	57	340	344	0.25	56	339	387	0.27
VAREIGVL	10,000	–	–	> 900	–	–	> 900	21	179	20	0.08	21	179	20	0.08

The output data reported show how the use of the Adaptive Truncation Criterion proposed in [1], enables to efficiently address the problem of “over-solving” the Newton equation, within linesearch-based truncated Newton methods. The adoption of this criterion could have important implications for future implementations of such methods, for solving large scale unconstrained optimization problems. Indeed, it leads to a noticeable reduction of the CG inner iterations, that is significant computational savings of the overall computational burden.
